# MiR-221 Influences Effector Functions and Actin Cytoskeleton in Mast Cells

**DOI:** 10.1371/journal.pone.0026133

**Published:** 2011-10-12

**Authors:** Ramon J. Mayoral, Lorenzo Deho, Nicole Rusca, Nenad Bartonicek, Harpreet Kaur Saini, Anton J. Enright, Silvia Monticelli

**Affiliations:** 1 Institute for Research in Biomedicine, Bellinzona, Switzerland; 2 EMBL - European Bioinformatics Institute, Wellcome Trust Genome Campus, Hinxton, Cambridge, United Kingdom; Oklahoma Medical Research Foundation, United States of America

## Abstract

Mast cells have essential effector and immunoregulatory functions in IgE-associated allergic disorders and certain innate and adaptive immune responses, but the role of miRNAs in regulating mast cell functions is almost completely unexplored. To examine the role of the activation-induced miRNA miR-221 in mouse mast cells, we developed robust lentiviral systems for miRNA overexpression and depletion. While miR-221 favored mast cell adhesion and migration towards SCF or antigen in trans-well migration assays, as well as cytokine production and degranulation in response to IgE-antigen complexes, neither miR-221 overexpression, nor its ablation, interfered with mast cell differentiation. Transcriptional profiling of miR-221-overexpressing mast cells revealed modulation of many transcripts, including several associated with the cytoskeleton; indeed, miR-221 overexpression was associated with reproducible increases in cortical actin in mast cells, and with altered cellular shape and cell cycle in murine fibroblasts. Our bioinformatics analysis showed that this effect was likely mediated by the composite effect of miR-221 on many primary and secondary targets in resting cells. Indeed, miR-221-induced cellular alterations could not be recapitulated by knockdown of one of the major targets of miR-221. We propose a model in which miR-221 has two different roles in mast cells: in resting cells, basal levels of miR-221 contribute to the regulation of the cell cycle and cytoskeleton, a general mechanism probably common to other miR-221-expressing cell types, such as fibroblasts. Vice versa, upon induction in response to mast cell stimulation, miR-221 effects are mast cell-specific and activation-dependent, contributing to the regulation of degranulation, cytokine production and cell adherence. Our studies provide new insights into the roles of miR-221 in mast cell biology, and identify novel mechanisms that may contribute to mast cell-related pathological conditions, such as asthma, allergy and mastocytosis.

## Introduction

Mast cells are cells of the innate immune system that reside in most tissues and are characterized by cytoplasmic granules containing active mediators such as histamine and proteases, which can be rapidly released upon activation. A broad panel of cytokines and chemokines is also rapidly synthesized upon acute stimulation, although some cytokines (such as TNFα) can be pre-stored in the granules [1], [2]. On the surface, mast cells are characterized by the expression of KIT and FcεRI (high-affinity IgE receptor). KIT, the receptor for the stem cell factor (SCF), is a critical regulator of mast cell differentiation, proliferation, activation and survival. Although mast cells have been considered for a long time detrimental to the host, mainly because of their major role as master effector cells in allergic responses, it is now becoming clear that depending on the context, mast cells can either positively or negatively regulate innate or adaptive immune responses to pathogens or allergens (for a recent review see [3]).

MicroRNAs (miRNAs) are small, non-coding RNAs that are emerging as major players in the regulation of endogenous gene expression in immune homeostasis [4]. Specifically, miRNAs regulate the expression of target genes by interacting with sites in the 3′ untranslated region (3′UTR) of their target mRNAs. MiRNAs regulate all aspects of a cell, including differentiation, function, proliferation, survival, metabolism, and responses to environmental changes. A single miRNA can potentially modulate the expression of hundreds of transcripts in a cell, both by direct and indirect effects, and as many as 90% of human genes may be regulated by miRNAs [5]. MiRNAs are implicated in various pathologic conditions as well as in tumorigenesis [4], [6], and within the immune system, deletion or overexpression of specific miRNAs can impair innate or adaptive immune responses [7].

While the importance of miRNAs in various types of normal and diseased cellular processes is by now well established, very little is known about the role of miRNAs in mast cell development, function and disease. Our lab identified miR-221/-222 as a family of miRNAs that is transcriptionally induced upon mast cell activation, and we showed that expression of miR-221 and/or miR-222 to levels similar to the endogenous of activated mast cells, led to reduced mast cell proliferation [8]. MiR-221 and miR-222 derive from the same primary transcript and share the same seed sequence, implying that they should recognize the same targets [9]. Following up on our previous studies, we investigated the role of miR-221 in mast cell differentiation and function. Specifically, we found that although miR-221 does not seem to affect mast cell differentiation, it has important roles in regulating multiple processes in differentiated mast cells, such as degranulation, adhesion and migration, some of which may be linked to a dysregulation in the actin cytoskeleton. Indeed, we found that alteration of miR-221 expression in mast cells and fibroblasts led not only to a reduction in cell proliferation similar to what we previously described using a different expression system, but also to an alteration of actin content and overall cellular shape in both cell types. Transcriptional profiling and bioinformatics analysis using the Sylamer algorithm [10], indicated that miR-221 effects in mast cells were mediated by the alterations of the level of expression of many primary and secondary targets. Importantly, such miR-221-mediated alterations of the cell phenotype could not be recapitulated by knockdown of one of the most prominent target for miR-221, suggesting that the observed effect of miR-221 in resting mast cells and fibroblasts is likely to be composite, due to the alteration of many genes. Moreover, we also observed mast cell-specific, activation-dependent effects of miR-221. Since miR-221 is expressed at basal level in mast cells, but it is also inducible upon stimulation, we propose a model in which miR-221 has a dual roles in these cells: at resting state, it contributes to the regulation of the cell cycle and cytoskeleton, a housekeeping effect that can be observed also in different cell types expressing this miRNA. However, in response to stimulation through IgE-antigen complexes, miR-221 effects are mast cell-specific and activation-dependent, contributing to the regulation of degranulation, cytokine production and cell adherence. Overall, our studies provide insights on the role of miRNAs in mast cells, and lay the groundwork for understanding some of the mechanisms underlying pathological conditions caused by mast cells, such as allergy and mastocytosis.

## Materials and Methods

### Ethics statement

All animal studies were performed in accordance with the Swiss Federal Veterinary Office guidelines and were approved by the Dipartimento della Sanita' e della Socialita', authorization number 18/2010.

### Plasmids

About 400bp of the mouse miR-221 or miR-222 genomic sequences were cloned into the pAPM lentiviral vector [11]. Point mutations in the seed sequence of miR-221 were introduced using the Quick Change Site-Directed Mutagenesis kit (Stratagene) and the following primers (mutations underlined): *miR-221mFW*: 5′-GTTTGTTAGGCAACATCGCGATTGTCTGCTGGGTTTCAGG; *miR-221mRV*: 5′- CCTGAAACCCAGCAGACAATCGCGATGTTGCCTAACAAAC. The control vector pAPM-shLuc, expressing a miR-30-based shRNA against luciferase, was obtained from Thomas Pertel and Jeremy Luban and contained the following sequence: 5′- CACAAACGCTCTCATCGACAAG. The miRNA target (miRT) vectors containing four sequences fully complementary to miR-221 and/or miR-222, were provided by Bernhard Gentner and Luigi Naldini [12].

### Cell cultures

For bone marrow-derived mast cells (BMMCs) differentiation, bone marrow cells from C57Bl/6 mice (6-8 weeks old) were differentiated and maintained in IMDM with 10% FBS, 2 mM L-glutamine, 0.1 mM non essential amino acids, 50 µMβ-mercaptoethanol, antibiotics and 50% WEHI-3 conditioned supernatant as a source of IL-3 [13], [14]. The IL-3-dependent mast cell line MC/9 (ATCC) was cultured as for primary BMMCs. 3T3 cells were cultured in DMEM supplemented with 10% FBS, 2 mM L-glutamine, 0.1 mM non-essential amino acids, 50 µMβ-mercaptoethanol and antibiotics.

### Transductions and transfections

Lentiviral transductions of mast cells were performed exactly as described [8], [13]. 3T3 cells were transduced with concentrated lentiviruses prepared exactly as described [8], [13] and selected with 2 µg/mL puromycin for several days before performing experiments. For transient transfections with oligonucleotides, 3T3 cells were transfected with Lipofectamine-2000 (Invitrogen) and 20pmol of siRNAs against p27^Kip1^ (siGENOME *Cdkn1b*) or control siRNAs (siGENOME non-targeting siRNA #2 and/or siGLO) (all from Thermo Scientific) following manufacturer's instructions. Cells were analyzed 48–72 h after transfection. Efficiency of transfection was assessed in each experiment by transfecting a fluorescent oligonucleotide (siGLO) and analyzing the percentage of siGLO+ cells by FACS, which usually ranged between 50 and 70%. The efficiency of protein knockdown was assessed by Western blot.

### RNA extraction and RT-PCR

Total RNA was extracted using TRIzol (Invitrogen) according to manufacturer's instructions. To analyze miRNA expression, qRT-PCR was performed using a miRNA reverse transcription kit and TaqMan miRNA assays from Applied Biosystems, following exactly manufacturer's instructions.

### Immunostainings and microscopy

Anti-KIT-APC, anti-FcεRIα-PE and anti-CD25-PE were purchased from eBioscience. For microscopy, 10^5^ BMMCs were labeled with CFSE (Invitrogen) and added to a monolayer of 3T3 cells on coverslips. After 12 hours, coverslips were washed to eliminate non-adherent cells, and cells were fixed with 3.7% p-formaldehyde, permeabilized with 0.1% Triton-X100 and stained with 160nM phalloidin-AlexaFluor-594 (Invitrogen). Glass slides were mounted using Gelvatol (20% polyvinyl alcohol, 100mM Tris-HCl pH 8.5, 2.5% DABCO). The same protocol was used to stain 3T3 cells grown directly on coverslips. Bright field images were captured with a Nikon Eclipse E800 and analyzed with the Openlab software (Improvision). For FACS-based quantification of F-Actin, 3·10^5^ cells were fixed 15–30 min in 4–5% p-formaldehyde, permeabilized 2min with 0.1% Triton-X100, and stained with phalloidin at a 1∶100 dilution.

### Degranulation, adhesion and migration assays

For degranulation assay, BMMCs (5·10^4^) were resuspended in 50 µL OptiMEM, 1% FBS and stimulated for 1 h with 1.5 µg/mL IgE-anti-DNP (clone SPE-7, Sigma) and 0.2 µg/mL DNP-HSA (Sigma). After stimulation, cell pellets were lysed in 50 µL of 0.5% Triton-X100 in OptiMEM, 1% FBS, and 50 µL of a 3.8 mM solution of the β-hexosaminidase substrate 4-nitrophenyl N-acetyl-β-D-glucosaminide (Sigma) were added to both cell lysates and supernatants. After incubation for 2 h at 37°C, the reaction was stopped with 90 µL glycine 0.2 M, pH 10.7, and absorbance was read at 405 nm. The percentage of degranulation was calculated as the ratio between the absorbance of supernatants and the total absorbance of supernatants and cell lysates [15]. Alternatively, degranulation was assessed using the same annexin V-PE kit used to detect apoptosis (BD-Pharmingen) [16], [17]. Briefly, cells were stimulated for 30 min with 1.5 µg/mL IgE-anti-DNP and 0.2 µg/mL DNP-HSA, in the presence or absence of 20 ng/mL SCF (Peprotech). Cells were then washed and stained with annexin V-PE following manufacturer's instructions. For adhesion experiments, 10^5^ BMMCs were added to a monolayer of 3T3 cells in 24-well plates. Floating cells were harvested 8h later, and adherent cells were detached using 10mM EDTA. In order to distinguish BMMCs from fibroblasts, cells were stained for KIT, and numbers of KIT+ cells were evaluated by FACS. The percentage of adherent cells was calculated as the ratio between adherent cells versus total. Cell migration was assayed using 24-well transwell chambers (Corning) with 8.0 µm pores in polycarbonate membranes. 2·10^5^ BMMCs were seeded in the upper chambers and allowed to migrate for 2 h. In some experiments, 20 ng/mL of SCF was used as chemoattractant, while in others BMMCs were first sensitized with 300 ng/mL IgE-anti-DNP for 12 h and then allowed to migrate towards 0.2 µg/mL DNP-HSA. The percentage of migrated cells was calculated as the ratio between the number of cells in the lower chamber versus total.

### Cell-cycle analysis and Western blots

For propidium iodide staining and DNA content analysis, non-confluent 3T3 cells were fixed in 70% ethanol for 45 min on ice, followed by incubation for 30 min at 37°C with 100 µg/mL RNaseA and 40 µg/ml propidium iodide. Cells were analyzed by FACS immediately afterwards. Total protein extracts for Western blot were prepared by cell lysis in Laemmli sample buffer. Samples were run on 12% SDS-polyacrylamide gels and immunodetection was performed with p27 C-19 and β-tubulin H-235 antibodies (Santa Cruz Biotechnologies).

### Microarrays and Sylamer analysis

Gene arrays were performed at Miltenyi Biotec using Agilent dual-color whole-genome oligo arrays and total RNA from three independent biological replicas. Genes found to be commonly regulated were functionally annotated. Sylamer analysis was performed through a Sylarray web server [18], [19]. Specifically, the Agilent probes were mapped to the 3′zUTRs of genes stored in the Sylarray database. The sequences were previously masked from low complexity regions and redundant UTR sequences with DUST algorithm and RSAT purge-sequence interface to Vmatch [20], [21]. As a control we also analyzed the mutant seed sequence to check for any evidence of its influence in the experiment and no significant association was found. Array data are MIAME compliant and are deposited in the MIAME compliant database Gene Expression Omnibus (GEO) with accession number GSE24462.

### Statistical analysis

Results are expressed as mean ± standard deviation. Comparisons were made using the Student's unpaired t-test and the GraphPad Prism Software.

### Supporting Information

Additional information can be found in the Supplementary Methods S1.

## Results

### A robust lentiviral system for manipulating microRNA expression in mast cells

**Figure 1:**
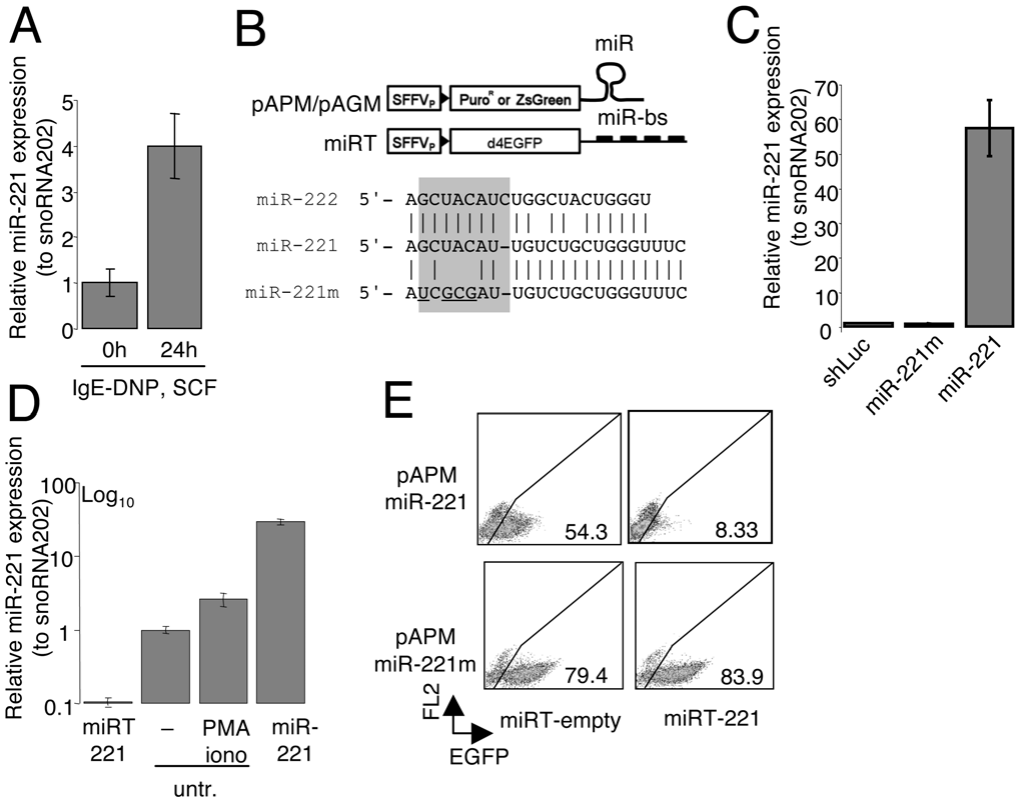
MiR-221 is upregulated upon mast cell activation and its expression levels can be altered using lentivirus-based systems. **A**) Differentiated BMMCs were either left resting or were stimulated with 1.5 µg/mL IgE anti-DNP, 0.2 µg/mL DNP-HSA and 20ng/mL SCF prior analysis of miR-221 expression by TaqMan qRT-PCR. SnoRNA202 was used as endogenous control. **B**) Schematic representation of the lentiviral vectors used to stably overexpress or functionally ablate miR-221 and miR-222 in BMMCs. Reporter genes were either puromycin or ZsGreen in the pAPM or pAGM vectors, respectively. Sequences corresponding to the mature murine miR-221, miR-222 and miR-221m are also shown. The miRT vectors contain 4 sequences fully complementary to miR-221 and/or miR-222 cloned downstream the reporter gene. SFFVp: spleen focus-forming virus promoter; d4EGFP: destabilized GFP; bs: binding sites. **C**) BMMCs were transduced with the control vector shLuc, miR-221 or miR-221-mutant. After selection with 2 µg/mL puromycin for 48h, miR-221 expression levels were assessed by TaqMan qRT-PCR. Expression levels are referred to the ones of the shLuc-transduced cells, which are set to one. **D**) BMMCs were transduced with miRT-221 (depleting) or miR-221 (overexpressing) vectors, and miR-221 expression levels were assessed by TaqMan qRT-PCR. Cells transduced with miR-221 were selected with 2 µg/mL puromycin for 48h prior analysis, while cells transduced with miRT-221 were sorted to >90% GFP+ prior RNA extraction. Untransduced cells were either left unstimulated or were stimulated with PMA and ionomycin for 24 h prior RNA extraction. Expression levels are referred to the untransduced and unstimulated cells, which are set to one. **E**) MC/9 cells were transduced with miR-221 (*upper panels*) or miR-221m (*lower panels*) and selected with 1 µg/mL puromycin prior a second transduction with the indicated miRT vectors (miRT-empty or miRT-221). GFP levels were determined by FACS. Transduction efficiency and levels of miR-221 expression or depletion are representative of tens of experiments, including all the experiments shown hereafter.

As we previously described for acute stimulation of mast cells [8], BMMCs stimulated with IgE-antigen complexes upregulated miR-221 expression (Figure 1A). While stimulation-dependent upregulation of this miRNA could be favored by SCF co-stimulation, SCF alone had no effect on miR-221 expression (not shown). To investigate the role of miR-221 in regulating primary mast cell functions, we developed a lentiviral system to manipulate miRNA expression in primary BMMC and used it to alter miR-221 expression. The pAPM/pAGM vectors were used to overexpress miR-221 or miR-222; as control, we used a mutant version of miR-221 (miR-221m), containing mutations in the seed region to abrogate target recognition, as well as a vector expressing an irrelevant hairpin (shLuc) (Figure 1B). The miR-221m mature sequence had no predicted targets as assessed by TargetScan [9]. The ‘miRNA target’ (miRT) vectors contain four miRNA binding sites (miR-bs) cloned downstream a GFP reporter gene, and they were used to functionally ablate miR-221/-222 [12]. Transcription from such vectors results in accumulation of decoy mRNAs that divert miRNAs from their physiological targets [22].

To assess expression from these vectors, BMMCs were transduced with the indicated vectors, and miRNA expression was assessed by qRT-PCR (Figures 1C and 1D). Compared to untransduced, unstimulated cells (expression set to 1 in Figures 1C and 1D), transduction of primary mast cells with pAGM/pAPM-miR-221 increased miR-221 expression by ∼60-fold, whereas transduction with miRT-221 decreased expression by ∼10-fold (Figure 1D). Transduction with the mutant miR-221m had no effect (Figure 1C). Initial experiments were performed using a vector (Tween) that induced only modest overexpression (∼4-fold), similar to the levels of endogenous miR-221 observed upon cell stimulation (Figure 1A) [8]. However, both types of vectors (weaker and stronger expression) gave similar results qualitatively, although the stronger vector provided bigger quantitative differences, and was therefore used in most of the subsequent experiments. To assess the functional effects of miRNA overexpression/ablation, the mast cell line MC/9 was transduced to overexpress miR-221 or the mutant miR-221m. Transduced cells were selected with puromycin, subjected to a second round of transduction with the miRT vectors, and monitored for GFP expression (Figure 1E). As a result of binding of the overexpressed miRNAs to their cognate sites in the 3′ UTR of the GFP reporter mRNA expressed from the miRT, GFP expression was strongly reduced specifically in cells expressing miR-221 but not the mutant miR-221m. We therefore used both validated systems (overexpression and ablation) to study mast cell differentiation in the presence or absence of miR-221.

MiR-221/-222 as well as the transcriptional repressor PLZF are both known important regulators of hematopoietic cell differentiation [23], [24], [25]. We previously showed that binding sites for PLZF were enriched in mast cell-specific DNaseI hypersensitive sites found upstream of the miR-221-222 genomic sequence [8]. To address the possible relation between PLZF and miR-221, we analyzed expression of both *Plzf* mRNA and miR-221 during mast cell differentiation (Supplementary Figure S1). We observed an inverse relation between *Plzf* and miR-221 expression during mast cell differentiation, and ectopic expression of PLZF in mast cells diminished miR-221 expression in response to acute stimulation, suggesting that PLZF is able to repress miR-221-222 induction either directly or indirectly, and possibly through PLZF-binding regulatory elements in the miR-221-222 locus [8]. However, ectopic expression of PLZF in differentiated mast cells had no effect on the basal levels of endogenous miR-221, indicating that other factors regulate basal expression of this miRNA in mast cells.

To assess whether miR-221/-222 may have a direct role in regulating the differentiation process in mast cells, we transduced bone marrow-derived hematopoietic progenitors with lentiviruses to either overexpress (pAPM) or ablate (miRT) miR-221 and/or miR-222 early during mast cell differentiation (Supplementary Figure S1). Differentiation was monitored over a period of at least three weeks by assessing the percentage of FcεRIα+ KIT+ cells. Interestingly, the percentage of BMMCs increased steadily over time in all samples, and mast cell differentiation was not significantly affected by either overexpression or ablation of miRNAs. Moreover, there was no obvious alteration in cell granularity or in the content of the granules (data not shown).

### MiR-221 regulates degranulation, migration and adherence in differentiated BMMCs

**Figure 2:**
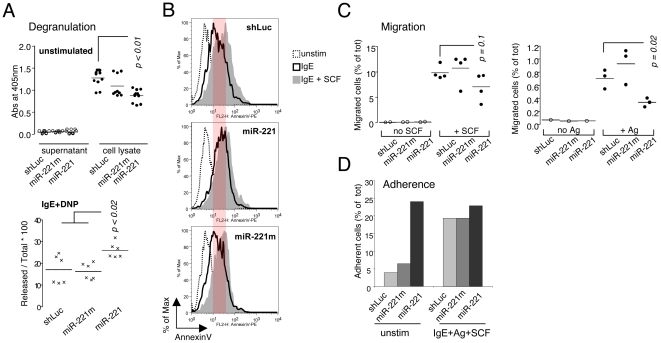
Degranulation, migration and adherence of cells overexpressing miR-221. **A**) Transduced BMMCs were either left unstimulated (*upper panel*; n = 5 experiments, each performed in triplicate) or were stimulated with IgE anti-DNP and DNP-HSA for 1h (*lower panel*; n = 2 experiments, each performed in triplicate) prior measurement of β-N-acetylhexosaminidase release to assess degranulation. **B**) Transduced BMMCs were either left unstimulated or were stimulated for 30min with IgE-antigen complexes alone or in combination with 20ng/mL of SCF. Cells were then stained with annexin V-PE to assess the extent of degranulation (representative of 2 independent experiments). The red shading in the figure indicates the difference between IgE versus IgE+SCF stimulation in control cell, which is reduced in miR-221-expressing cells). **C**) *Right panel*: BMMCs transduced with indicated vectors were seeded in the upper well of a transwell chamber and then allowed to migrate towards the lower chamber containing no chemoattractant (empty circles) or SCF (black circles). Each circle represents one independent experiment. *Left panel*: same as above, except that cells were sensitized with anti-DNP IgE overnight, and allowed to migrate towards the antigen (DNP-HSA). **D**) BMMCs transduced with the indicated vectors were seeded on a monolayer of 3T3 cells, and were either left resting or were stimulated for 8h with IgE-antigen complexes and SCF. Cells were then detached by treatment with 10mM EDTA, and the percentage of adherent cells versus total was assessed by surface staining for KIT and FACS analysis. Shown is one experiment out of at least six.

Since there was no effect of miR-221 in mast cell differentiation, we set out to investigate its role in mast cell functions, especially the ones connected to signaling through the FcεRI, given that miR-221 expression is inducible upon stimulation. Differentiated BMMCs were lentivirally transduced to force expression of miR-221, followed by analysis of the effects on mast cell degranulation, migration and adherence (Figure 2). Upon activation, mast cells release an array of enzymes that are pre-stored in cytoplasmic granules. We analyzed the ability of miR-overexpressing BMMCs to degranulate upon stimulation using β-hexosaminidase activity in the supernatant of activated cells as a measure of degranulation (Figure 2A). In resting conditions, cells did not degranulate, regardless of miRNA expression (Figure 2A, *top panel*) but, upon stimulation with IgE and antigen, BMMCs overexpressing miR-221 degranulated more compared to the controls (Figure 2A, *lower panel*), although they also showed a slightly reduced content of β-hexosaminidase in the granules to begin with (Figure 2A, *top panel*). To further confirm these results and to assess the effect of different conditions of stimulation (namely IgE crosslinking with or without SCF co-stimulation), we assessed degranulation of cells overexpressing miR-221 or controls by using a staining with annexin V. This staining takes advantage of the fact that mast cells do not die upon stimulation (which is instead a survival factor [26]) and that during the membrane fusion process of degranulation, annexin V binding occurs at sites of secretory granule exposure to the cell surface [16], [17]. In general, BMMCs stimulated with a combination of IgE-antigen and SCF degranulated more strongly compared to cells stimulated without SCF (Figure 2B). However, compared to the controls (shLuc and miR-221m) miR-221 overexpression increased degranulation in response to IgE, as shown already by β-hexosaminidase assay, but miR-221-overexpressing cells did not further degranulate in response to the combination of both SCF and IgE crosslinking (middle panel). This could be due to the fact that the SCF receptor KIT is expressed at lower levels on these cells (see below), or to the fact that in the presence of miR-221 cells are activated more strongly upon IgE crosslinking, and cannot be further activated by the combination of IgE and SCF.

Next, we investigated the capability of BMMCs to migrate in a transwell system (Figure 2C). We found that cells overexpressing miR-221 migrated significantly less towards SCF as compared to the controls (Figure 2C, *right panel*). KIT is a target for miR-221 [24], therefore, to understand whether the reduced migration was due to an intrinsic feature of cells overexpressing miR-221, or to a reduced ability to ‘sense’ SCF in the environment due to lowered expression of KIT, we repeated the same experiment sensitizing BMMCs with IgE-anti-DNP prior inducing migration towards DNP-HSA (Figure 2C, *left panel*). The discernible, albeit modest, migration of control-transduced cells towards the antigen was significantly impaired if miR-221 was overexpressed, indicating that the reduced migration was due to effects of miR-221 on targets other than KIT.

Another process promoted by the stimulation through the FcεRI is adherence of mast cells to the substrate. Since mature mast cells do not normally circulate *in vivo*, but reside in tissues, we explored whether miR-221 had any role in regulating cell adherence and migration, as these are essential processes not only under normal homeostatic conditions, but also during inflammation and tumorigenesis. As *in vitro*-differentiated mast cells grow in suspension, we assessed the ability of BMMCs to adhere to a feeder layer of fibroblasts in a co-culture system (Figure 2D). Increased adherence is a normal process observed upon stimulation of mast cells with IgE and antigen, however, BMMCs expressing miR-221 adhered at a higher percentage compared to the controls even in resting, unstimulated conditions. Vice versa, upon stimulation all cells were able to adhere to the feeder layer of fibroblasts at comparable levels, regardless of miRNA expression. Since miR-221 overexpression was sufficient by itself to increase adherence, our data indicate that endogenous miR-221/-222 upregulation upon cell activation may contribute to the increased adherence of mast cells observed upon stimulation.

These results may point towards a role for miR-221 in regulating the signaling cascade originating from the FcεRI. In this context, it is important to highlight that miR-221 overexpression did not alter surface expression of FcεRI (see below). To evaluate whether signaling from the FcεRI could be affected in presence of miR-221, we assessed the levels of ERK phosphorylation in response to IgE crosslinking (Supplementary Figure S2). In all conditions and time-points tested, there was no significant difference in ERK phosphorylation in cells overexpressing miR-221 compared to the controls, suggesting that miR-221 might not affect directly the signaling cascade from the FcεRI. However, in miR-221-expressing cells, in addition to increased degranulation (Figure 2, panels A and B), we also observed increased cytokine production (IL-6 and TNFα) in response to IgE crosslinking, but not to LPS (Supplementary Figure S2 and data not shown). This observation may indicate that miR-221 expression favors mast cell activation in response to IgE-antigen complexes, however in a way that doesn't seem to grossly affect ERK phosphorylation.

### Microarray analysis identified genes affected by miR-221

**Figure 3:**
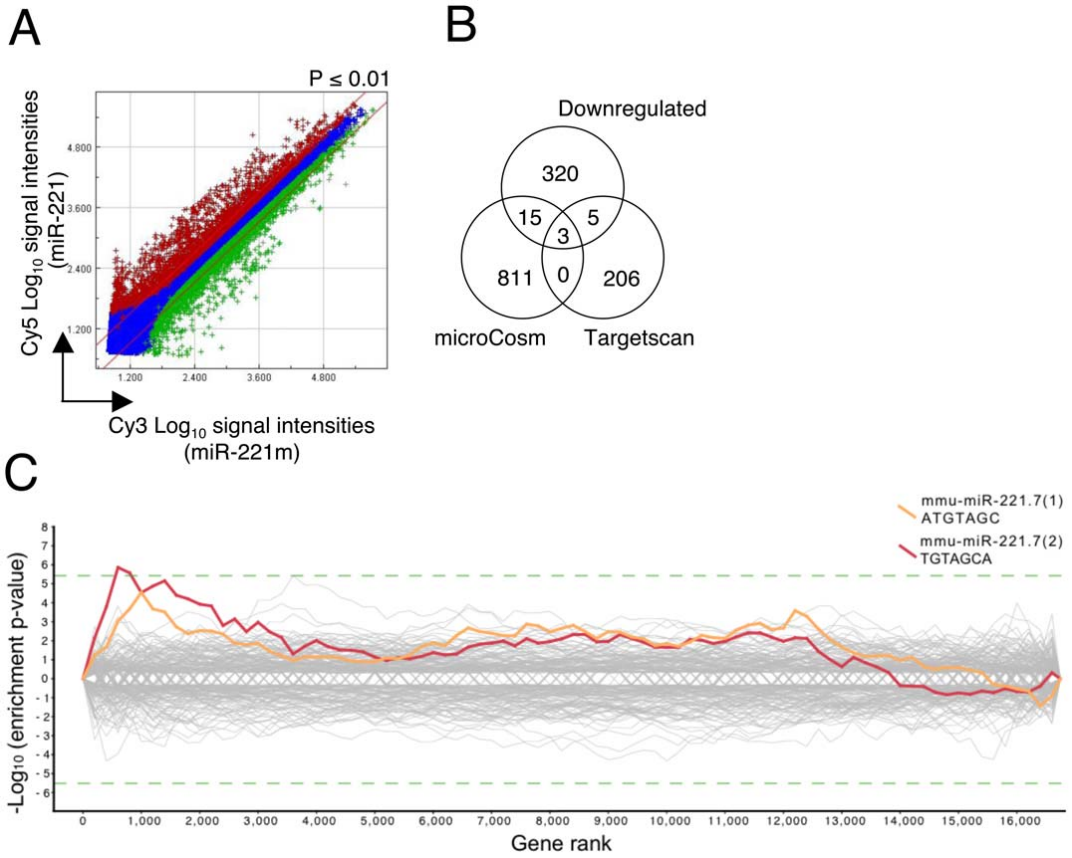
Identification of genes dysregulated upon miR-221 overexpression in BMMCs. **A**) Double-log scatter plot comparing the differential expression of mRNAs in BMMCs transduced with the miR-221 and miR-221m. Red diagonal lines define the areas of 2-fold differential signal intensities. Blue cross: unchanged genes. Red cross: significantly upregulated genes (p-value <0.01). Green cross: significantly downregulated genes (p-value <0.01). **B**) Comparison between significantly downregulated genes and genes predicted to be miR-221 targets by microCosm and Targetscan. **C**) Sylamer plot analysis for the Seed Complementary Region (SCR) words corresponding to the seed of miR-221 (red) and its one-nucleotide shifted sequence (orange). Log_10_- transformed and sign-adjusted enrichment P values for each SCR word, relative to P values of all other words, are plotted on the Y-axis, against the ranked gene list on the X-axis (left, downregulated genes; right, upregulated genes). The dashed green line defines the Bonferroni corrected p-value cutoff for significance of 0.05.

To gain insight into the mechanisms underlying such pleiotropic effects of miR-221 in mast cells, we performed a microarray analysis of BMMCs overexpressing miR-221 or miR-221m, as it has been reported that indeed the impact of a miRNA on protein production can be closely approximated using mRNA arrays [27]. Out of ∼42.000 transcripts analyzed, we found 397 significantly upregulated genes in miR-221-expressing BMMCs, as well as 343 significantly downregulated genes as compared to cells transduced with miR-221m (Figure 3A). Importantly, the known miR-221 targets *Cdkn1b* (p27^Kip1^) and *Kit* were found to be downregulated with a mean fold-change repression of -3.2 and -2.0, respectively. While the upregulated genes presumably reflect secondary changes due to changes in the expression of primary targets, the downregulated gene subset may contain both primary targets as well as secondary effects.

Of note, when we compared the list of our downregulated genes with the targets predicted by TargetScan [9] and microCosm [28], we found very few overlapping genes, which could indicate either that only few downregulated genes are the real primary targets, or that many targets are relevant only in the specific mast cell context (Figure 3B). To better understand how miR-221 could regulate gene expression specifically in mast cells, we therefore performed a Sylamer analysis [19] on the complete gene list from the arrays, which was ranked from most downregulated to most upregulated (left to right in the graph in Figure 3C). Sylamer is an algorithm for determining whether specific 6, 7 or 8nt motifs corresponding to miRNA seed sequences are enriched or depleted in a ranked gene list [19]. The Sylamer plots can be used to analyze influence of a miRNA on an expression set profile. Sylamer calculates enrichment of seed words in 3′UTRs of a sorted gene list, providing a p-value for genes left of each rank versus the genes on the right. In this case, the upper left portion of the plot represents an influence of miRNAs on downregulated genes (Figure 3C), indicating a specific influence of miR-221 on a subset of downregulated genes. Any point of a line provides a p-value that a word is enriched in the UTRs left of it on the X-axis. Although in some cases most of the effect of a given miRNA in a particular cellular context goes primarily through one or few molecular targets, in other cases this does not provide the complete picture of miRNA effect on the transcriptome and proteome of a cell [27], [29], [30]. Our array analysis showed that the transcriptomes of miR-221-overexpressing cells differed by the expression of 740 genes compared to the controls, and the Sylamer analysis indicated that many of the downregulated genes in mast cells were likely to be directly affected by miR-221 expression, suggesting that the effect of this miRNA goes through the fine modulation of a multitude of targets.

**Figure 4:**
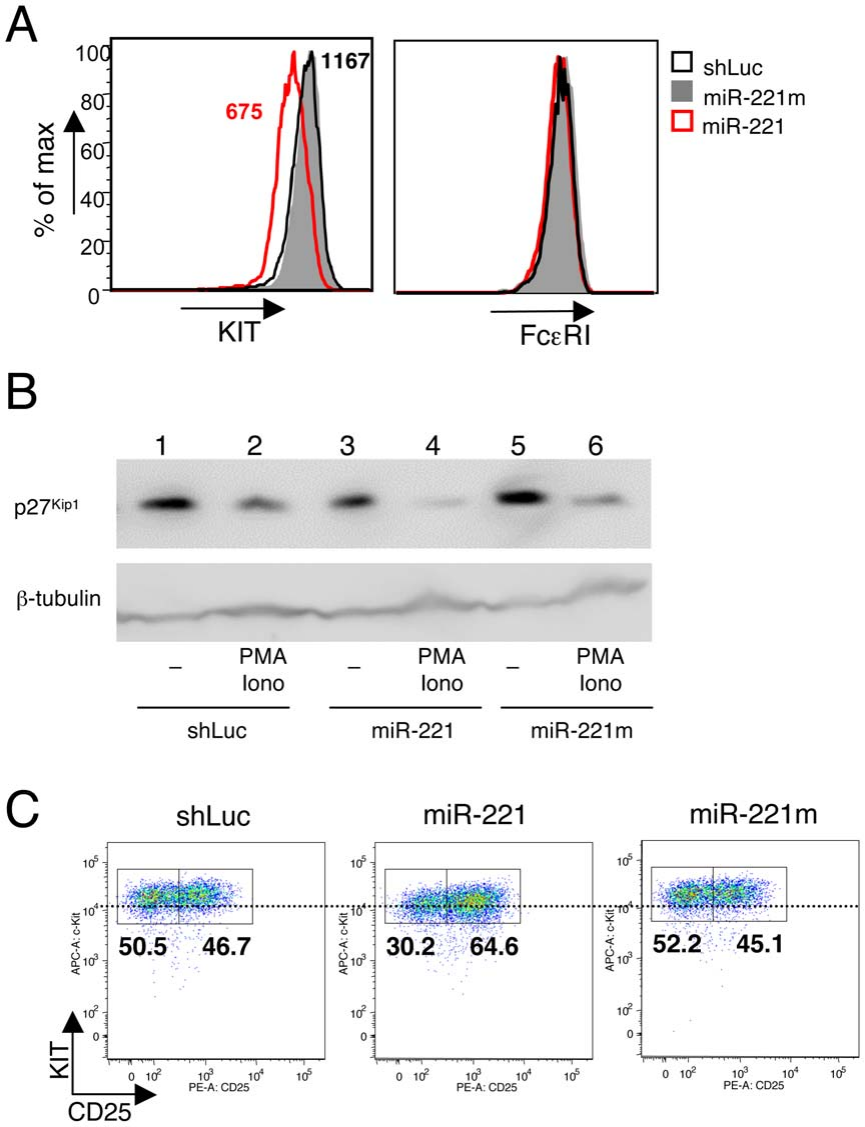
MiR-221 primary and secondary targets are altered also at protein level. **A**) Differentiated BMMCs were either left untransduced or were transduced with the indicated vectors. After selection with puromycin, surface expression of KIT (*left*) and FcεRI (*right*) was assessed by FACS. The mean fluorescence intensity (MFI) for KIT expression in miR-221- (red) and shLuc- (black) expressing cells is also indicated. **B**) Differentiated BMMCs as in A) were either left untreated or were stimulated with 20nM PMA and 1 µM ionomycin for 24h prior lysis and Western blot analysis of p27^Kip1^ expression. β-tubulin was used as loading control. **C**) BMMCs as in A) were stained and FACS-analyzed to assess surface expression of KIT and CD25. The horizontal dotted line was added as a reference to appreciate the expected KIT dowregulation in miR-221 expressing cells.

Some of the targets that were up- or downregulated were also confirmed at protein level; among others, we selected two of the already known targets for miR-221, *Kit* and *Cdkn1b* [8], [24], [31], as well as one upregulated gene (*Il2Ra*) that is particularly relevant in mast cell biology as a marker for systemic mastocytosis [32], [33]. As assessed by surface staining, levels of KIT, but not of FcεRI, were significantly diminished (∼2-fold) in BMMCs overexpressing miR-221 (Figure 4A). In contrast, as we previously described [8], levels of p27^Kip1^ protein were especially decreased in stimulated cells (Figure 4B, compare lane 4 with lanes 2 and 6). While CD25 (*Il2Ra*) is normally not expressed by human mast cells in non-pathogenic conditions [32], [33], it is normally expressed by a subset of *in vitro*-differentiated murine BMMCs (Figure 4C) as well as by a subset of peritoneal and tissue mast cells in the mouse (Deho' and Monticelli, unpublished observation). The mean fold increase for *Il2Ra* from the arrays was 2.1, which strongly correlated with the increased surface expression of this marker in cells overexpressing miR-221 (Figure 4C).

Although some of the targets for miR-221 were individually confirmed, our arrays experiments and Sylamer analysis indicated that miR-221 determines the downregulation of more than 200 primary targets and the subsequent secondary alterations of many genes in the transcriptome. Therefore, to gain insights into the function of the genes that showed altered expression in the presence of miR-221, we performed functional grouping and gene ontology (GO) analysis, and found that many basic biological processes were affected (Table 1). Among the downregulated genes, the categories that were statistically significant were surprisingly few, and belonging for the most part to very general metabolic pathways, such as protein folding, transcription, lipid metabolism and cell differentiation. This finding may suggest that in resting, unstimulated cells, miR-221/-222 regulate basic metabolic processes that are common to many different cell types and/or species, and correlates with the fact that these miRNAs belong to a family very conserved in evolution, down to at least zebrafish [8]. Although they are most likely due to secondary changes in the transcriptome, the GO categories of the upregulated genes included several pathways that we also observed altered experimentally, such as cell proliferation, cell adhesion and cell migration. Among these latter GO categories, we focused our attention on one of the most statistically significant one, namely the cytoskeleton (which was also present in the GO list of terms for the downregulated genes, although with a p-value of 0.1), as we hypothesized that an alteration in the ability of the cells to properly regulate the cytoskeleton could to some extent explain why these miRNAs affect many different biological processes, at least in unstimulated cells.

**Table 1:** Gene Ontology (GO) frequency distribution for downregulated and upregulated genes.

DOWNREGULATED – Categories	Times Observed	Probability
Cell migration	15	5.30E-05
Protein folding/modification	30	1.80E-04
Transcription	38	5.70E-04
Lipid metabolism	17	2.30E-03
Cell differentiation	28	3.20E-03
Other metabolism	29	8.00E-03
**UPREGULATED – Categories**	**Times Observed**	**Probability**
Receptor signaling	73	9.40E-09
Cell proliferation	26	4.60E-08
Cytoskeleton	35	4.70E-08
Cell cycle	27	6.40E-08
Cell adhesion	26	3.30E-07
Cell differentiation	37	1.10E-05
Kinase/Phosphatase signaling	23	1.30E-05
Other metabolism	39	1.90E-05
Protein folding/modification	35	2.50E-05
Development	46	2.90E-05
G-protein signaling	43	8.80E-05
Cell migration	15	3.10E-04
Nucleotide metabolism	26	3.70E-04
Inflammation	11	1.00E-03
Intracellular trafficking	20	5.08E-03

Gene ontology (Bonferroni corrected) of mRNAs that were downregulated or upregulated in the microarrays. A cutoff of P = 0.01 was used for the categories to be considered statistically significant.

### MiR-221-dependent alterations of the cytoskeleton and actin content in mast cells and 3T3 cells

**Figure 5:**
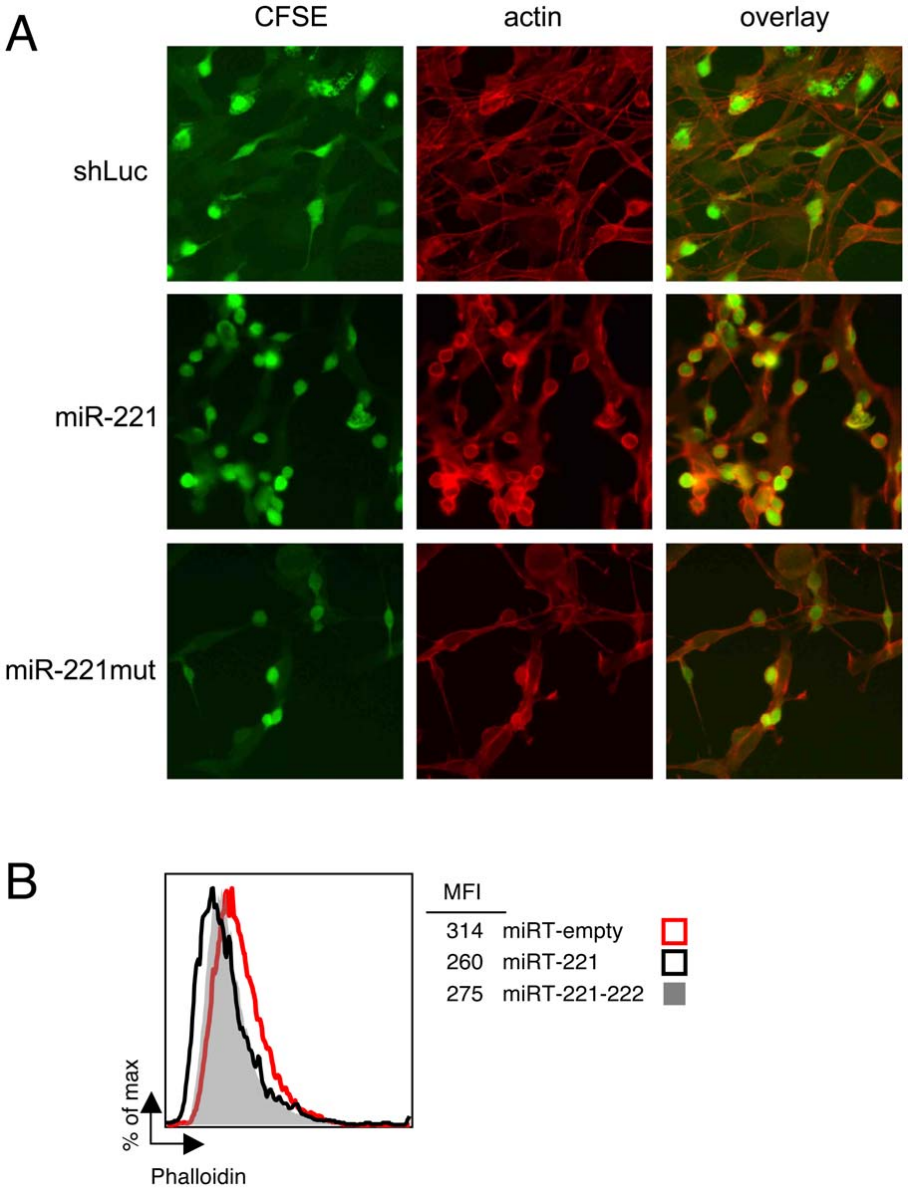
MiR-221 influences BMMCs actin cytoskeleton. **A**) Transduced BMMCs were loaded with CFSE (green) and allowed to adhere to a monolayer of 3T3 cells. After washing to remove non-adherent cells, F-actin was stained with phalloidin-AlexaFluor-594 (red) prior imaging with a fluorescence microscope. Shown is one representative experiment out of three. **B**) BMMCs were transduced with the indicated miRT vectors, and overall F-Actin content was assessed by FACS staining with phalloidin. Shown is one representative experiment out of three.

To assess whether miR-221 expression could affect the cytoskeleton, we stained transduced BMMCs with phalloidin (Figure 5A). We used the same co-culture system with fibroblasts used in Figure 2D to assess cell adherence, and to distinguish mast cells from the underlying layer of feeder cells (also stained by phalloidin), BMMCs were first labeled with CFSE. Strikingly, not only miR-221-overexpressing cells showed increased numbers of adherent cells (as shown also in Figure 2D), but while the actin ring underneath the plasma membrane was barely visible in control cells, cells overexpressing miR-221 (or miR-222, not shown) showed the presence of a much thicker ring (Figure 5A). Moreover, when we quantified the overall cellular amount of F-actin in cells depleted for miR-221 (using the miRT-depleting vectors), we observed a small but reproducible decrease in the amount of F-actin present in these cells (Figure 5B), further indicating that these miRNAs might be important regulators of the actin organization in mast cells.

**Figure 6:**
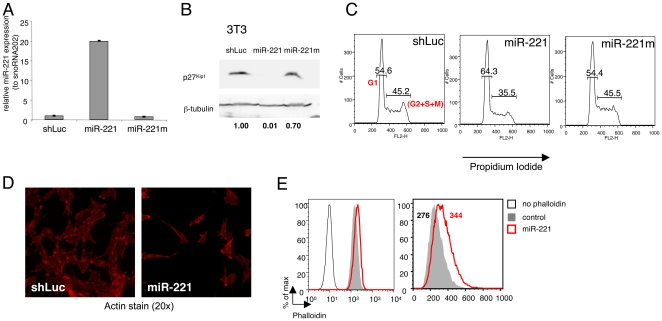
MiR-221 affects cell proliferation and actin cytoskeleton in fibroblasts. **A**) 3T3 cells were transduced with lentiviral vectors to express either miR-221 or controls shLuc and miR-221m. After selection with 2 µg/mL for at least 2 days, total RNA was extracted and levels of miR-221 expression were assessed by TaqMan qRT-PCR. SnoRNA202 was used as endogenous control; for comparison, levels of miR-221 expression in the shLuc-transduced sample were set to one. Representative of two independent experiments. **B**) 3T3 cells as in A) were lysed in Laemmli sample buffer and p27^Kip1^ expression was analyzed by Western blot. Expression of β-tubulin was used for normalization in the quantification of bands intensity (numbers below the blot). One representative experiment out of 3 is shown. **C**) Cells as in A) were collected during exponential-phase growth and the DNA content was assessed with propidium iodide staining as a measure of the number of cells in each stage of the cell cycle. For simplicity, only the percentage of G1 cells (left in each plot) versus the percentage of dividing cells (S+G2+M) are shown. One out of three independent experiments is shown. **D**) Cells as in A) were seeded on a coverslip and allowed to adhere overnight, after which they were fixed in 4% paraformaldehyde and stained with phalloidin-AlexaFluor-594 prior imaging with a fluorescent microscope. Shown is one representative experiment out of two. **E**) Cells as in A) were trypsinized to single cell suspension, fixed and stained as in B) and analyzed by FACS. The left plot shows (on a log scale) the unstained control compared to stained samples, while on the left the scale was changed to linear to better show the differences among samples. The MFI for each sample is also provided. Depending on the experiment, control cells were either untransduced cells, cells transduced with shLuc- or the miR-221m-expressing vector, or all of the above. Shown is one representative experiment out of four.

To independently confirm these results, and to investigate whether the observed effect was a general feature of this miRNA or a cell type-specific effect due to alterations of targets relevant only in the mast cell context, we transduced 3T3 fibroblasts with the same lentiviral vectors used on mast cells. 3T3 cells expressed low levels of endogenous miR-221 that were increased ∼20-folds upon transduction with a miR-221 expressing vector (Figure 6A). MiR-221 overexpression in 3T3 cells led to a strong downregulation of endogenous p27^Kip1^, even more remarkable than the one observed in mast cells (Figure 6B). Despite such strong downregulation of the cell-cycle inhibitor p27^Kip1^, 3T3 cells overexpressing miR-221 showed the same reduced proliferation that we previously described for mast cells (Figure 6C) [8]. Moreover, 3T3 cells overexpressing miR-221 showed overall altered morphology, with odd, elongated and/or irregular shapes (Figure 6D), as well as a slightly increased content of F-actin (Figure 6E), indicating that the miR-221-dependent effects on the cytoskeleton and cell cycle observed in resting mast cells are likely to be due to the dysregulation of targets that are ubiquitously expressed and are therefore cell type-independent. However, FcεRI stimulation led to mast cell-specific (or at least not present in fibroblasts) effects of miR-221, with increased degranulation and cytokine production.

**Figure 7:**
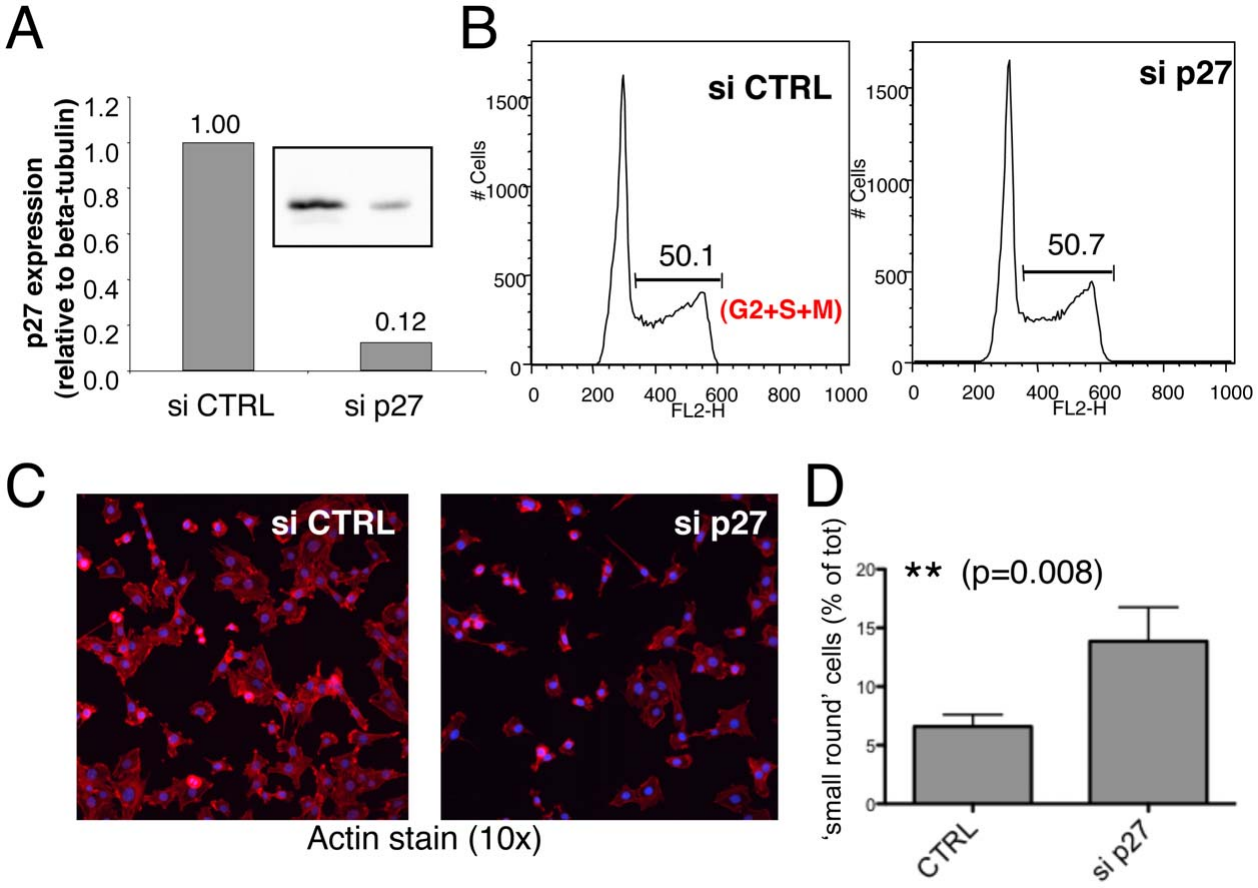
Knockdown of the miR-221 target p27^Kip1^ does not recapitulate the composite effects of miR-221 expression. **A**) 3T3 cells were transfected with siRNA oligonucleotides against p27^Kip1^ (si p27) or with a non-targeting control (si CTRL). 48–72 h later cells were collected and lysed for Western blot analysis. Shown is the quantification (against β-tubulin, used as loading control) of one representative blot; the actual blot for p27^Kip1^ is shown in the inset. Efficiency of transfection ranged between 50–70% depending on the experiment, with a downregulation of protein expression as assessed by Western blot that ranged widely between 0.05–80% of the levels of control, with the latter case (highest expression, minimal knockdown) showing no phenotypic effect. **B**) 3T3 cells as in A) were trypsinized to single-cell suspension, fixed in ethanol and stained with propidium iodide for cell-cycle analysis. **C**) Cells as in A) were seeded on coverslips and allowed to adhere overnight prior fixation with 4% paraformaldehyde and staining with phalloidin-AlexaFluor-594. Nuclei were counterstained with DAPI (blue). **D**) Quantification of the percentage of ‘small, round’ cells observed in an experiment as in C). For each sample, 8 random fields were counted, at a magnification of 10x.

Analyzing the data from our transcriptome profiling, we found that in the ‘cytoskeleton’ group of downregulated genes, the top candidate, most downregulated gene was *Cdkn1b* (p27^Kip1^), and specifically the one splice variant that can be regulated by miR-221/-222 (as we previously described in [8]). While p27^Kip1^ is a cell cycle inhibitor with a well established role in cell cycle progression at the G1-S transition, it has also been shown that cytoplasmic p27^Kip1^ plays an important role in cell motility and migration, and that p27^Kip1^-deficient fibroblasts fail to form long cellular protrusions, assume an overall rounded shape [34] and show reduced migration [35]. To assess whether miR-221-dependent down-regulation of p27^Kip1^ may have a role in regulating 3T3 and mast cells shape and cytoskeleton, we therefore performed a knockdown of p27^Kip1^ in 3T3 cells using siRNAs (Figure 7). Efficiency of transfection and p27^Kip1^ knockdown were evaluated by transfection and FACS analysis of a fluorescent ds-oligo (siGLO) and by Western blot, respectively (data not shown and Figure 7A). It has to be noted that the efficiency of transfection was at the most ∼70%, so that the residual protein observed in Western blot may in part be due to the fact that some cells still expressed significant levels of p27^Kip1^. However, the knockdown of p27^Kip1^ did not alter the overall cell-cycle profile of 3T3 cells (Figure 7B), and the cells did not show any particularly altered shape, apart from a slight increase in the percentage of cells that were smaller and more rounded (Figure 7, panels C and D). Although this effect was fairly modest (even in the experiments with the strongest downregulation of p27^Kip1^ only <15% of the cells were counted as ‘small and round’, compared to the controls), it was in line with what was previously reported for *Cdkn1b*-deleted fibroblasts. Indeed, p27^Kip1^ KO fibroblasts were shown to have a rounded shape with no alterations in the cell cycle [34]. Most importantly, the knockdown of p27^Kip1^ did not recapitulate the phenotype we observed in miR-221 overexpressing 3T3 cells, as cell cycle and cellular shape were either unaltered or completely different from what we observed in miR-221-transduced cells, suggesting (as indeed indicated by our Sylamer analysis), that the effect of this miRNA is composite and goes through the down-modulation of multiple targets.

Although the mechanisms underlying the role of miR-221 specifically in mast cells in both resting and stimulated conditions will require further investigation and will be the subject of future work, our data show that the effect of this miRNA goes through the alteration of the levels of many targets in the mast cell transcriptome, that it has important roles in regulating mast cell physiology, and finally that at least some of its biologic effects in resting cells may be explained by alterations in the actin cytoskeleton of mast cells.

## Discussion

Although mast cells have a long lifespan, accumulation of a large mast cell burden *in vivo* is usually not observed. Thus, a homeostatic mechanism must exist to limit differentiation and accumulation of mast cells in peripheral tissues, both during basal maintenance, and during mast cell hyperplasia in inflammatory processes [36]. MiR-221 is a likely candidate as a regulator of mast cell functions: we previously showed that it is transcriptionally induced upon mast cell activation, and that it contributes to the modulation of proliferation in unstimulated mast cells [8]. We now showed that miR-221 may have more ubiquitous effects to fine-tune proliferation and actin cytoskeleton in cells as different as resting mast cells and fibroblasts. On the other hand, miR-221 also influenced many other features of differentiated mast cells, including cytokine production, migration, adhesion and survival upon withdrawal of essential cytokines (Supplementary Figure S3), all mechanisms that may be involved in regulating tissue accumulation and resolution of hyperplasia upon eradication of inflammation *in vivo*.

While we were able to show that at least some of these effects may be linked to a miR-221-dependent regulation of the actin cytoskeleton in mast cells, this miRNA may have both ‘housekeeping’ functions, active in different cell types, like the regulation of the cytoskeleton and cell cycle, but also cell-specific effects on targets such as KIT and CD25 that are expressed by mast cells but not fibroblasts. As for targets, our arrays data and bioinformatics analysis showed that expression of miR-221 led to the downregulation of 343 genes, many of which are likely to be primary targets, containing the miR-221 seed-complementary sequence in their 3′UTR. For this reason, we think that the effect of miR-221 in resting mast cells may be composite and due to small alterations of many genes in the transcriptome. However, since we did not perform knockdown of every individual potential target, the possibility remains that the effect of miR-221 in mast cells and fibroblasts might be mediated by the downregulation of one or few predominant genes that we were so far unable to identify. Yet, although levels of p27^Kip1^ in miR-221 overexpressing fibroblasts were very low, the individual knockdown of this protein led to a phenotype that somewhat resembled the phenotype of p27^Kip1^-deleted fibroblasts, but did not resemble in any way the phenotype of miR-221 overexpressing cells, suggesting that miR-221 target regulation is more complex then the downregulation of one predominant target.

While it is clear that miRNAs regulate many different cellular processes, understanding the details of the functions of individual miRNAs remains challenging. Indeed, as in the case of miR-221 and -222, miRNAs are frequently present as families of redundant genes. MiR-221 and -222 share the same seed sequence (Figure 1B), and should recognize the same targets [9]; although most of the experiments shown here were performed primarily with miR-221, we also performed experiments using miR-222-expressing vectors, which always gave results similar to miR-221, both in mast cells and in fibroblasts (not shown). Another difficult aspect of dealing with miRNAs is that each miRNA has many potential targets with disparate functions, with no means to decide a priori which is the most meaningful and therefore worthy of experimental validation. Finally, the degree of target downregulation is typically less than 50% [29], [37], and understanding which fraction of the miRNA:target interaction is actually relevant for a biological response remains a challenging task. In our hands, p27^Kip1^ was a striking example of this phenomenon, as while it was downregulated very strongly upon miR-221 expression, its knockdown did not recapitulate the complex effect of miR-221 expression.

Although clearly able to regulate some general cellular features, such as cell cycle and cytoskeleton in resting mast cell, we speculate that miR-221/-222 may also be part of a mechanism that contribute to mediate many of the changes that occur in mast cells upon stimulation. Specifically, miR-221 may have a ‘housekeeping’ function in resting mast cells, where it is expressed at low, basal levels, and contributes to the regulation of the cell cycle and cytoskeleton. Vice versa, miR-221 is also transcriptionally activated upon stimulation, and in this case it would contribute to the regulation of cell-type specific, FcεRI-dependent mechanisms, such as cytokine production, degranulation and cell adhesion. In this perspective, it is important to highlight that although transcription of *pri-miR-221-222* starts early upon IgE stimulation, mature miR-221 accumulation occurs with a ‘slow’ kinetic (the peak of mature miRNA expression is reached at ∼24h after initial stimulation) [8]. We therefore speculate that the slow kinetic of accumulation of the mature form of miR-221 may actually contribute to a resting, but ‘activation-ready’ cellular state, that favors increased degranulation, adherence and cytokine production upon challenge (Supplementary Figure S4).

Indeed, mast cell stimulation normally leads to increased adherence and increased survival of the cells, which can be further activated by a secondary challenge [26], [38]. Upon secondary encounter with the antigen mast cells respond with increased degranulation and cytokine production (reviewed in [38]). Although such higher levels of degranulation and cytokine production are usually considered to be the result of increased levels of FcεRI, the fact that miR-221-expressing cells showed no perturbation of FcεRI expression and at the same time increased adherence (even in the absence of stimulation), increased degranulation as well as cytokine production suggests that miR-221 may contribute to such intensified cellular response upon secondary challenge. The processes mediated by this miRNA may also have a role in regulating mast cell homeostasis in tissues and possibly also in pathologic conditions. It will be therefore interesting to assess what is the role of miR-221/-222 *in vivo* in mouse models, both in terms of cell homeostasis at steady-state or upon sensitization and challenge with an antigen or with a pathogen, and this will be the subject of future work.

Along the line of a possible role of these miRNAs in mast cell-related diseases, we showed that miR-221 and miR-222 regulate levels of KIT expression in mast cells. Of note, KIT levels are often abnormally low in patients with indolent systemic mastocytosis, although the D816V gain-of-function mutation in KIT is present in most of these patients and is sufficient to cause indolent mastocytosis [39]. This downregulation of KIT expression is even more pronounced in poor prognosis systemic mastocytosis (i.e. systemic mastocytosis associated with a clonal hematopoietic disease, aggressive systemic mastocytosis and mast cell leukemia) [39], suggesting that multiple layers of regulation of this receptor may be acting in diseased cells. In this context, it will be interesting to assess whether miR-221/-222 could be part of a molecular mechanism involved in KIT regulation specifically in mastocytosis. Indeed, although the D816V mutation may be sufficient to cause indolent mastocytosis, other additional defects, that remain to be identified, are required to induce aggressive mast cell disorders, and may very well include dysregulated miRNA expression [40], [41], [42]. This is even more intriguing considering that CD25, which is so far the best available diagnostic marker for systemic mastocytosis with bone marrow involvement [32], [33], is also regulated by these miRNAs. While miR-221/-222 were already implicated in various human cancers for their effect on proliferation, we show here for the first time that these miRNAs also regulate mast cell adhesion, migration, and survival, all processes that may have implications in mastocytosis.

In summary, although miR-221 doesn't seem to affect mast cell differentiation, it influences many features of the biology of differentiated cells. Specifically, at basal levels, in resting conditions, these effects are likely to be linked, at least in part, to the regulation of the actin cytoskeleton and cell cycle, two features that are regulated by miR-221 independently of the cell type. However, upon mast cell stimulation, miR-221 may have some more cell type-specific, activation-dependent effects, influencing the extent of degranulation, adherence and cytokine production in response to IgE-antigen complexes. Although still speculative, we propose a model in which miR-221 would have two different roles in mast cells: in resting cells, it contributes to normal cell homeostasis through the regulation of the cell cycle and cytoskeleton, while upon induction following acute stimulation, it contributes to increase the strength of the response to antigenic challenge. Overall, our work provides new insights into previously unknown effects of miR-221 in mast cell biology, and may have important implications for our understanding of the molecular mechanisms underlying normal and pathologic mast cell conditions.

## Supporting Information

Figure S1 **MiR-221 expression can be regulated by the transcriptional repressor PLZF, but it has no role in BMMC differentiation.** **A**) Lineage depleted (Lin–, lacking surface expression of CD5, CD45R, CD11b, Gr-1, 7-4 and Ter-119) and Lin+ bone marrow cells were either immediately used for RNA extraction or differentiated to mast cells in IL-3 containing medium [43]. Total RNA from Lin– derived mast cells was used to assess *Plzf* mRNA expression (*upper panel*) and miR-221 (*lower panel*). **B**) Differentiated BMMCs were lentivirally transduced to ectopically express PLZF. After puromycin selection for 48h, cells were either left untreated or were stimulated with 20nM PMA and 1 µM ionomycin for 24h, prior RNA extraction and analysis of *Plzf* and miR-221 expression. **C**) Lin– cells were transduced with the indicated vectors to either force (pAPM) or ablate (miRT) miR-221 expression, and were cultured for three weeks in the presence of IL-3 to allow mast cell differentiation. Cultures were analyzed weekly for the presence of mast cells (FcεRIα+ KIT+) by surface staining. Each point represents one independent experiment. Cells transduced with shLuc, miR-221 and miR-221m vectors were selected with 2 µg/mL puromycin, while cells transduced with the miRT vectors (empty, T-221 and T-221-222) were FACS-sorted for GFP expression. **D**) Total RNA was extracted from cells treated as in C) at the end of the differentiation period (percentage of FcεRIα+ KIT+ cells was greater than 90%), and expression of miR-221 was assessed by TaqMan qRT-PCR. SnoRNA202 was used as endogenous control, with levels of miR-221 expression set to one in the shLuc-transduced sample. Cells transduced with shLuc and miR-221 vectors were selected with 2 µg/mL puromycin, while cells transduced with the miRT vectors (empty, T-221 and T-221-222) were FACS-sorted for GFP expression.
(TIF)

Figure S2 **MiR-221 expression does not significantly alter ERK phosphorylation in mast cells, but favors cytokine production.** **A**) Differentiated BMMCs transduced with the indicated vectors were sensitized with 1.5 µg/mL of IgE-anti-DNP for 15min on ice. After washing to remove unbound IgE, 200ng/mL of DNP-HSA were added, and the cells were immediately moved to a 37°C water bath for 5, 15 and 45min. Cells were subsequently fixed, permeabilized and stained with biotinylated anti-phospho-p44/42 MAPK (Erk1/2). The mean fluorescence intensity for each sample is indicated on the left. **B**) Cells as in A) were either left untreated or were stimulated for 5min with 1 µM ionomycin and 20nM PMA at 37°C, after which cells were fixed, permeabilized and stained with an anti-Erk1/2 antibody. The mean fluorescence intensity for each sample is indicated next to the histograms. Shown is one experiment out of two. **C)** BMMCs as in A) were stimulated with 1.5 µg/mL IgE-anti-DNP and 200ng/mL DNP-HSA for 3.5h at 37°C. To block export from the Golgi, brefeldin A (10 µg/mL) was added in the last two hours of stimulation. Cells were subsequently fixed, permeabilized and stained with anti-IL-6-PE and anti-TNF-α-PE-Cy7. One representative experiment out of three is shown.
(TIF)

Figure S3 **MiR-221 expression favors mast cell survival in response to withdrawal of essential cytokines.** BMMCs were transduced with the indicated vectors and differentiated in the presence of IL-3 only (*top panel*) or IL-3 + 10ng/mL SCF (*bottom panel*) for three weeks, after which all cytokines were washed out of the culture medium for at least 24h prior evaluation of early cell death with annexin V and 7AAD staining. Shown is the percentage of cells in early apoptosis (annexin V+ 7AAD–).
(TIF)

Figure S4 **A ‘dual’ role for miR-221 in mast cells.** Speculative model of the possible roles of miR-221 in mast cells. At resting state, basal levels of miR-221 expression would regulate homeostatic mechanisms such as the cell cycle and cytoskeleton. These effects are not necessarily cell type-specific, as they can be active also in fibroblasts, which also express miR-221. Upon mast cell activation, ‘early’ effects include the release of preformed mediators from the cytoplasmic granules and the *de novo* synthesis of other mediators, including a broad panel of cytokines. The peak of accumulation of mature miR-221 is instead a ‘late’ event upon cell stimulation, and we speculate that it may contribute to the strength of the response upon secondary challenge, with increased degranulation, cytokine production and cell adherence.
(TIF)

Methods S1 (DOC)
