# Supplementary figures and images for: MiR-221 Influences Effector Functions and Actin Cytoskeleton in Mast Cells
Source: PLoS One. 2011 Oct 12;6(10):e26133. doi: 10.1371/journal.pone.0026133 (PMC3192147; doi:10.1371/journal.pone.0026133)

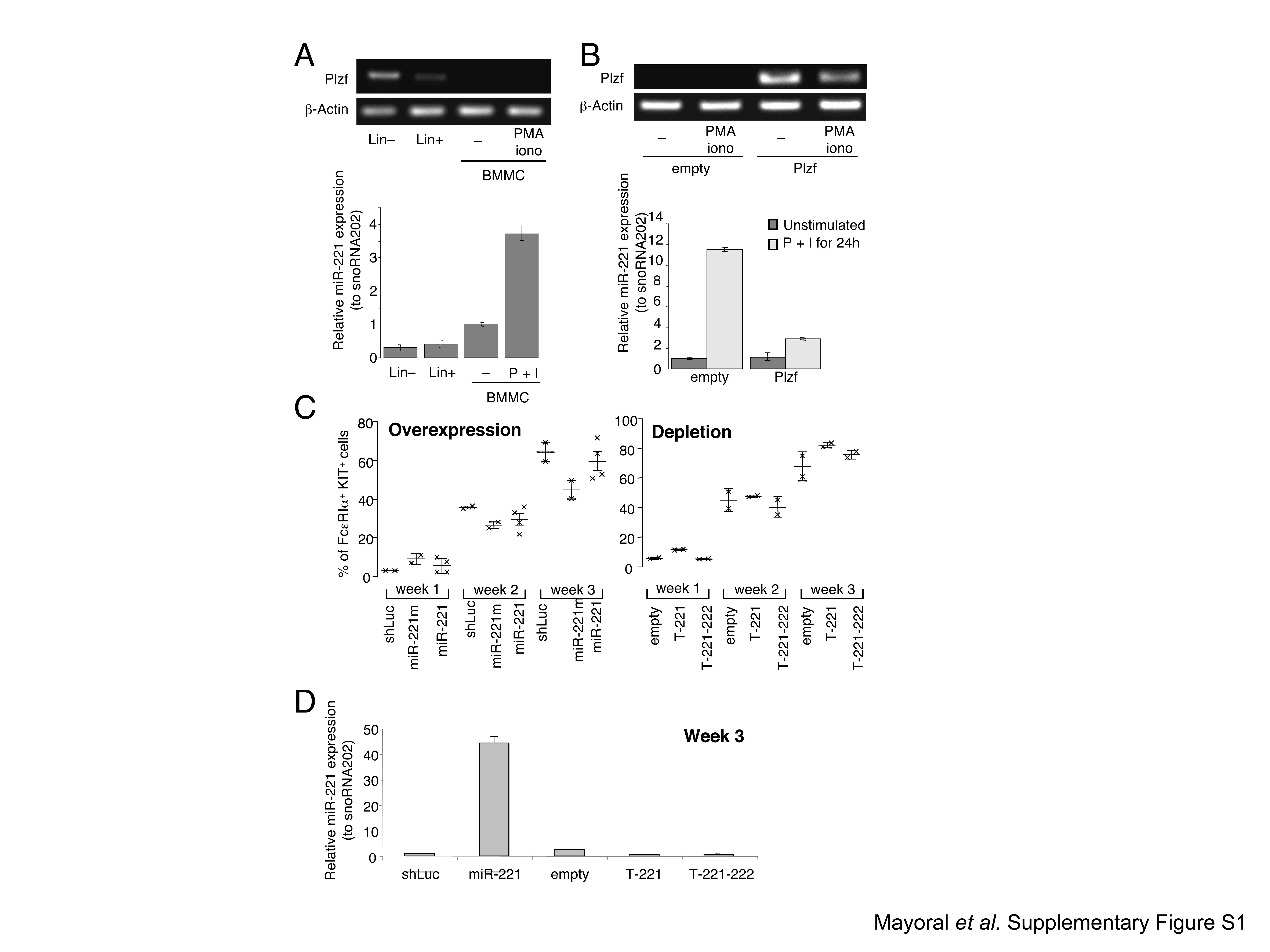

Supplement: Figure S1 — MiR-221 expression can be regulated by the transcriptional repressor PLZF, but it has no role in BMMC differentiation. A) Lineage depleted (Lin–, lacking surface expression of CD5, CD45R, CD11b, Gr-1, 7-4 and Ter-119) and Lin+ bone marrow cells were either immediately used for RNA extraction or differentiated to mast cells in IL-3 containing medium [43]. Total RNA from Lin– derived mast cells was used to assess Plzf mRNA expression (upper panel) and miR-221 (lower panel). B) Differentiated BMMCs were lentivirally transduced to ectopically express PLZF. After puromycin selection for 48h, cells were either left untreated or were stimulated with 20nM PMA and 1 µM ionomycin for 24h, prior RNA extraction and analysis of Plzf and miR-221 expression. C) Lin– cells were transduced with the indicated vectors to either force (pAPM) or ablate (miRT) miR-221 expression, and were cultured for three weeks in the presence of IL-3 to allow mast cell differentiation. Cultures were analyzed weekly for the presence of mast cells (FcεRIα+ KIT+) by surface staining. Each point represents one independent experiment. Cells transduced with shLuc, miR-221 and miR-221m vectors were selected with 2 µg/mL puromycin, while cells transduced with the miRT vectors (empty, T-221 and T-221-222) were FACS-sorted for GFP expression. D) Total RNA was extracted from cells treated as in C) at the end of the differentiation period (percentage of FcεRIα+ KIT+ cells was greater than 90%), and expression of miR-221 was assessed by TaqMan qRT-PCR. SnoRNA202 was used as endogenous control, with levels of miR-221 expression set to one in the shLuc-transduced sample. Cells transduced with shLuc and miR-221 vectors were selected with 2 µg/mL puromycin, while cells transduced with the miRT vectors (empty, T-221 and T-221-222) were FACS-sorted for GFP expression. (TIF) [file pone.0026133.s001.tif]

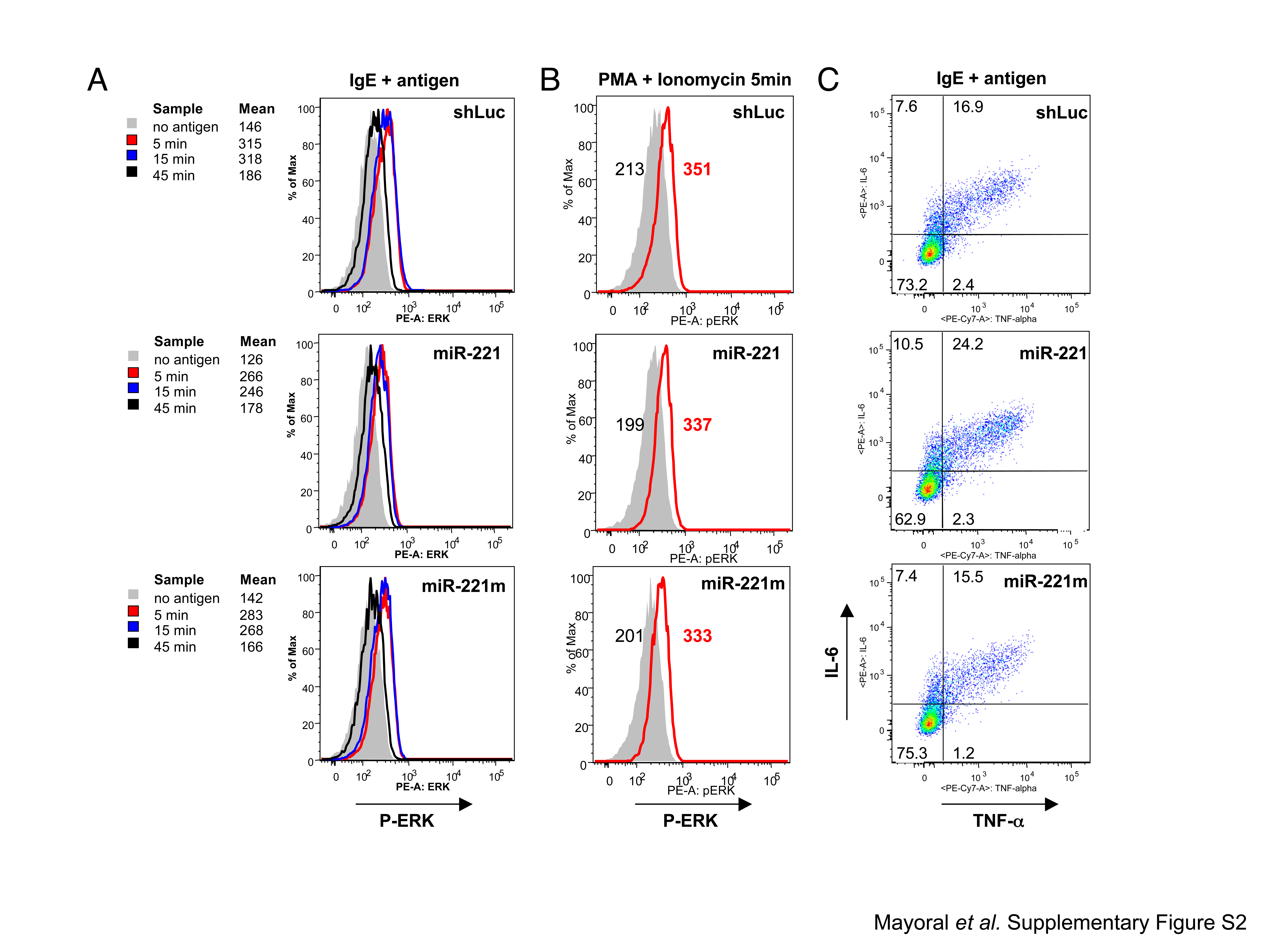

Supplement: Figure S2 — MiR-221 expression does not significantly alter ERK phosphorylation in mast cells, but favors cytokine production. A) Differentiated BMMCs transduced with the indicated vectors were sensitized with 1.5 µg/mL of IgE-anti-DNP for 15min on ice. After washing to remove unbound IgE, 200ng/mL of DNP-HSA were added, and the cells were immediately moved to a 37°C water bath for 5, 15 and 45min. Cells were subsequently fixed, permeabilized and stained with biotinylated anti-phospho-p44/42 MAPK (Erk1/2). The mean fluorescence intensity for each sample is indicated on the left. B) Cells as in A) were either left untreated or were stimulated for 5min with 1 µM ionomycin and 20nM PMA at 37°C, after which cells were fixed, permeabilized and stained with an anti-Erk1/2 antibody. The mean fluorescence intensity for each sample is indicated next to the histograms. Shown is one experiment out of two. C) BMMCs as in A) were stimulated with 1.5 µg/mL IgE-anti-DNP and 200ng/mL DNP-HSA for 3.5h at 37°C. To block export from the Golgi, brefeldin A (10 µg/mL) was added in the last two hours of stimulation. Cells were subsequently fixed, permeabilized and stained with anti-IL-6-PE and anti-TNF-α-PE-Cy7. One representative experiment out of three is shown. (TIF) [file pone.0026133.s002.tif]

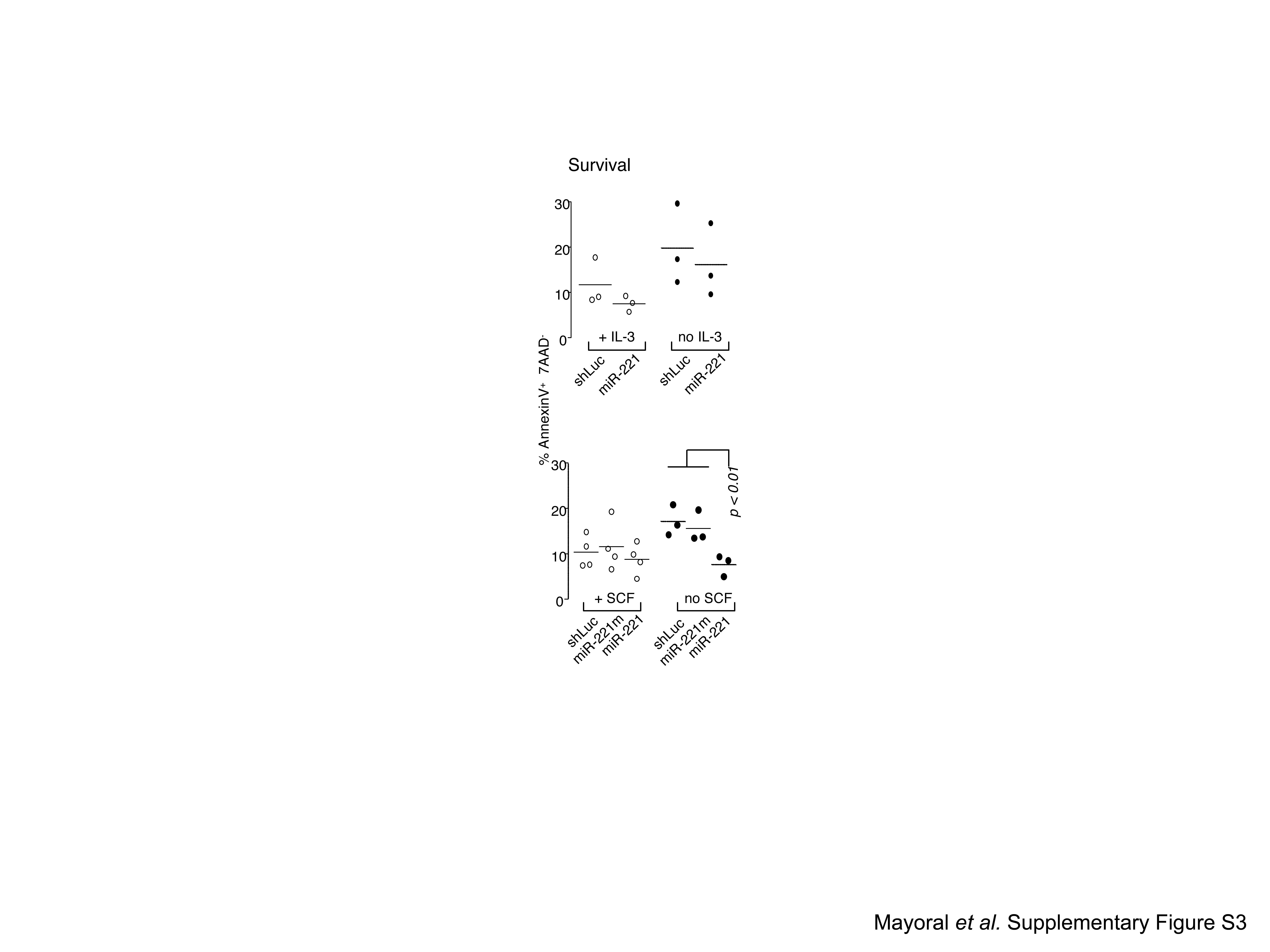

Supplement: Figure S3 — MiR-221 expression favors mast cell survival in response to withdrawal of essential cytokines. BMMCs were transduced with the indicated vectors and differentiated in the presence of IL-3 only (top panel) or IL-3 + 10ng/mL SCF (bottom panel) for three weeks, after which all cytokines were washed out of the culture medium for at least 24h prior evaluation of early cell death with annexin V and 7AAD staining. Shown is the percentage of cells in early apoptosis (annexin V+ 7AAD–). (TIF) [file pone.0026133.s003.tif]

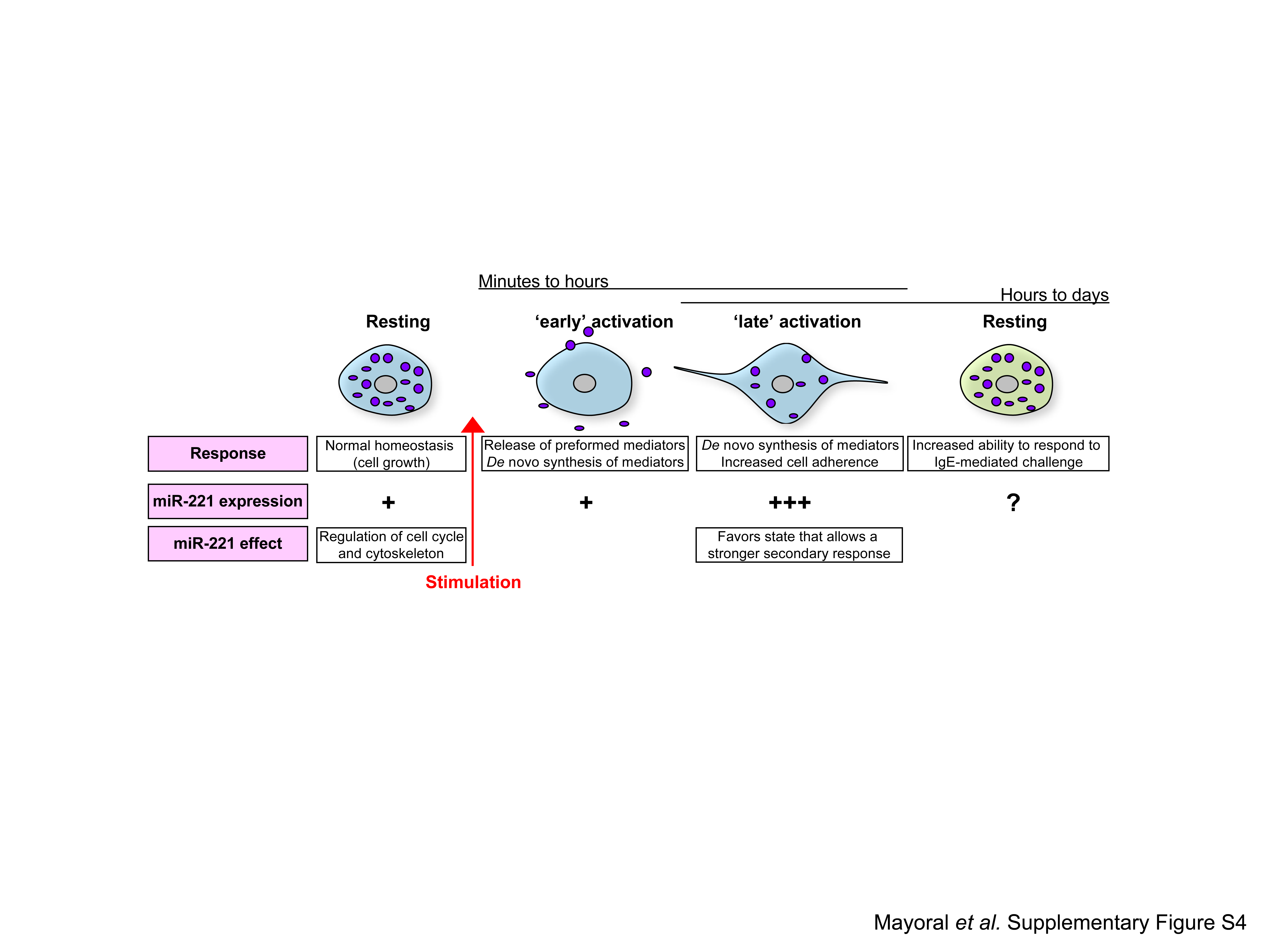

Supplement: Figure S4 — A ‘dual’ role for miR-221 in mast cells. Speculative model of the possible roles of miR-221 in mast cells. At resting state, basal levels of miR-221 expression would regulate homeostatic mechanisms such as the cell cycle and cytoskeleton. These effects are not necessarily cell type-specific, as they can be active also in fibroblasts, which also express miR-221. Upon mast cell activation, ‘early’ effects include the release of preformed mediators from the cytoplasmic granules and the de novo synthesis of other mediators, including a broad panel of cytokines. The peak of accumulation of mature miR-221 is instead a ‘late’ event upon cell stimulation, and we speculate that it may contribute to the strength of the response upon secondary challenge, with increased degranulation, cytokine production and cell adherence. (TIF) [file pone.0026133.s004.tif]
