# Supplementary material for: MiR-221 Influences Effector Functions and Actin Cytoskeleton in Mast Cells
Source: PLoS One. 2011 Oct 12;6(10):e26133. doi: 10.1371/journal.pone.0026133 (PMC3192147; doi:10.1371/journal.pone.0026133)
Supplement: Methods S1 — (DOC) [file pone.0026133.s005.doc]

**Supporting Information**

**Supplementary Methods**

**Plasmids.** Murine Plzf was provided by Wilfried Ellmeier [1], and it was subcloned into the scALPS vector [2] using standard cloning techniques.

**Cell cultures.** Lineage-negative (Lin–) cells [3] were enriched from bone marrow using a lineage-depletion kit (Miltenyi Biotec) and cultured for 48h in serum-free media (StemCell Technologies) containing 100ng/mL SCF, 100ng/mL Flt3L, 50ng/mL TPO, 20ng/mL IL-3 (all from Peprotech), antibiotics; media was then changed to regular BMMC media.

**RT-PCR.** For semi-quantitative RT-PCR, total RNA (1μg) was reverse transcribed using the iScript kit (Bio-Rad), before PCR amplification using the following primers: *PlzfFW*: 5’- CCTTTGTGTGTGATCAATGCGGTG; *PlzfRV*: 5’- TGGTGCTTGAGGCTGAACTTCTTG; *β-actinFW*: 5’- GCAGCTCCTTCGTTGCCGGT; *β-actinRV*: 5’- GGCTTTGCACATGCCGGAGC.

**Apoptosis detection.** Apoptosis was evaluated using the annexin V-PE apoptosis detection kit (BD-Pharmingen) following exactly manufacturer’s instructions.

**Intracellular staining.** For intracellular cytokine staining, cells were stimulated with 1.5μg/mL IgE anti-DNP and 200ng/mL DNP-HSA in complete media for 3,5h at 37°C. Brefeldin-A (10μg/mL, Sigma) was added in the last 2h of stimulation. Cells were then fixed in 4% paraformaldehyde for 10min at RT, and permeabilized with 0.5% saponin, 1% BSA in PBS, prior staining with fluorochrome-conjugated anti-cytokines antibodies (anti-IL-6, anti-TNF-α, both from eBioscience) for 30min at RT. For intracellular staining of phospho-ERK, cells were sensitized with 1.5μg/mL of IgE-anti-DNP for 15min on ice. After washing to remove unbound IgE, 200ng/mL of DNP-HSA were added, and the cells were immediately moved to a 37°C water bath for an incubation time of 5, 15 and 45min. Alternatively, cells were stimulated with 1μM ionomycin and 20nM PMA at 37°C. Cells were subsequently fixed in 4% paraformaldehyde, permeabilized with 0.5% saponin, 1% BSA in PBS and stained intracellularly with biotinylated anti-phospho-p44/42 MAPK (Erk1/2) (Cell Signaling).

**Supplementary References**

1. Raberger J, Schebesta A, Sakaguchi S, Boucheron N, Blomberg KE, et al. (2008) The transcriptional regulator PLZF induces the development of CD44 high memory phenotype T cells. Proc Natl Acad Sci U S A 105: 17919-17924.

2. Neagu MR, Ziegler P, Pertel T, Strambio-De-Castillia C, Grutter C, et al. (2009) Potent inhibition of HIV-1 by TRIM5-cyclophilin fusion proteins engineered from human components. J Clin Invest 119: 3035-3047.

3. Kondo M, Wagers AJ, Manz MG, Prohaska SS, Scherer DC, et al. (2003) Biology of hematopoietic stem cells and progenitors: implications for clinical application. Annu Rev Immunol 21: 759-806.
